# A Comparative Study of the Properties of Recycled Concrete Prepared with Nano-SiO_2_ and CO_2_ Cured Recycled Coarse Aggregates Subjected to Aggressive Ions Environment

**DOI:** 10.3390/ma14174960

**Published:** 2021-08-31

**Authors:** Song Gao, Yaoyao Gong, Nan Li, Shunli Ban, Ang Liu

**Affiliations:** 1 School of Civil Engineering, Qingdao University of Technology, Qingdao 266011, China; gaosong727@126.com (S.G.); gongyy1202@163.com (Y.G.); c18637829796@163.com (S.B.); 2 Collaborative Innovation Center of Engineering Construction and Safety in Shandong Blue Economic Zone, Qingdao Technological University, Qingdao 266033, China; 3 Liaoning Province Chaoyang Ecology and Environment Monitoring Center, Chaoyang 122000, China; linan31@163.com

**Keywords:** RCA, carbonization, nano-SiO_2_, interface transition zone, ion attack

## Abstract

This research focused on the modification effects on recycled concrete (RC) prepared with nano-SiO_2_ and CO_2_ cured recycled coarse aggregates (RCA) subjected to an aggressive ions environment. For this purpose, RCA was first simply crushed and modified by nano-SiO_2_ and CO_2,_ respectively, and the compressive strength, ions permeability as well as the macro properties and features of the interface transition zone (ITZ) of RC were investigated after soaking in 3.5% NaCl solution and 5% Na_2_SO_4_ solution for 30 days, respectively. The results show that nano-SiO_2_ modified RC displays higher compressive strength and ions penetration resistance than that treated by carbonation. Besides, we find that ions attack has a significant influence on the microcracks width and micro-hardness of the ITZ between old aggregate and old mortar. The surface topography, elemental distribution and micro-hardness demonstrate that nano-SiO_2_ curing can significantly decrease the microcracks width as well as Cl^−^ and SO_4_^2−^ penetration in ITZ, thus increasing the micro-hardness, compared with CO_2_ treatment.

## 1. Introduction

Large amounts of construction waste are being produced from China’s urbanization process [1]. Waste concrete is the most suitable raw material for renewable building materials among construction waste [2]. In recent years, the use of recycled coarse aggregates (RCA) has attracted particular attention because it can not only save natural aggregates but effectively reduce the environmental pollution caused by waste concrete [3]. Therefore, it has become a research hotspot to popularize waste concrete to produce RCA and apply it to RC.

**Figure 1:**
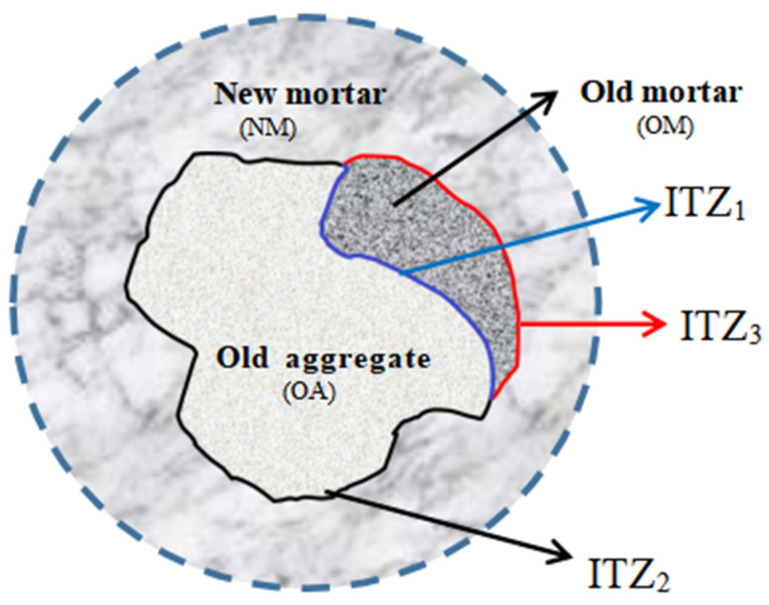
Diagram of multiple interface areas.

RCA is a composite of original natural aggregate and old cement mortar formed by crushing, screening, and processing waste concrete [2]. This is because of the existence of old mortar on the surface, compared with ordinary concrete, which contains more complex interface transition zones [4] (ITZs), as shown in Figure 1 (ITZ_1_: the interface between old aggregate and old mortar; ITZ_2_: the interface between old mortar-new mortar; ITZ_3_: the interface between old aggregate and new mortar). Loose porous ITZs are considered to be regenerated internal defects, which affects mechanical properties of RC [5]. In addition, the ITZs of concrete are more likely to become the accepted passage of the erosive medium, which degrade the concrete durability [6,7]. In recent years, some researchers have proposed a range of modification methods to improve the properties of recycled concrete (RC) and RCA. Among these, accelerated carbonation treatment has been increasingly reported due to the facile operation and improved quality of RCA. Wang et al. [8] studied the carbonation treatment (28 d) on the influence of micro-hardness and ITZ of RCA. The results show that the micro-hardness values of the three interface zones were all increased, and the width of the transition zone decreased in the carbonized RC samples treated for 28 days. Bao et al. [9] carbonized RCA and characterized the ITZ of RC. They found that a large amount of calcium carbonate could be precipitated on the surface of RCA by carbonization treatment, which could further react with aluminate in new mortar to form monocarbonate. Recently, nano materials (e.g., nano-SiO_2_) modification technology were also utilized to strengthen the quality of RC. Yan [10] and Yang [11] used different soaking mechanisms to soak RCA with nano-SiO_2_ and obtained consistent results. Nano-SiO_2_ soaking can significantly improve the performance of recycled aggregate, and significantly improve the macro mechanical properties of RC. Zeng et al. [12] found that nano-modification can improve the protection and corrosion cracking resistance of concrete reinforcement by soaking RCA with nano-SiO_2_. Xiao et al. [13] used nano-SiO_2_ to pre-soak RCA, and found that after modification, the apparent density and crushing index of RCA remained basically unchanged, and the water absorption decreased by 1.28%. In addition Qiu et al. [14] studied the influencing factors of microbial mineralization and sedimentation modification of RCA and found that bacterial species, bacterial concentration, pH value, temperature, aggregate characteristics have an influence on microbial mineralization and sedimentation, and the microbial mineralization and sedimentation rate of RCA can be improved by reasonably controlling the culture and sedimentation conditions.

At present, treatment by accelerated carbonation or nano-SiO_2_ has been considered to be an effective measure to enhance the properties of RCA and RC. However, research on the difference of the modification effects under aggressive ions environment between the two curing strategies, especially the micro-mechanism between ITZ is rarely conducted. Therefore, the objective of the present work is to compare the repair mechanism of such two treatment methods as well as the microstructure and properties evolution rules of RC under different aggressive ions environment, and to establish the relationship between remediation, erosion and performance.

## 2. Materials and Experimental Details

### 2.1. Raw Materials and Mixture Ratio

**Figure 2:**
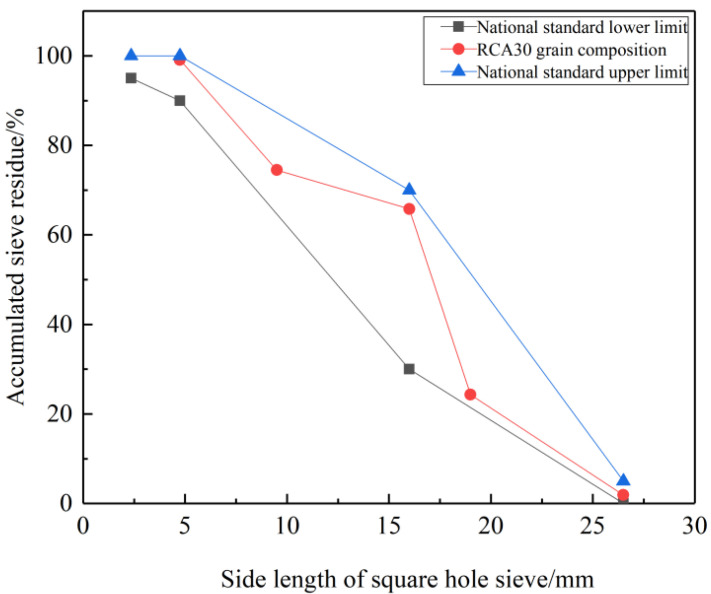
Grain composition.

RCA: obtained from the original concrete with C30 strength grade after standard curing for 28 days according to the standard for test methods of mechanical properties of ordinary concrete (GB/T50081-2002), then crushed and screened by a jaw crusher. Its particle size is between 4.75 mm and 26.5 mm, and its grain composition is shown in Figure 2, which meets the requirements of RCA quality evaluation standard (GB/T25177-2010). Among them, the cement used in the original concrete is ordinary Portland cement of P.O. 42.5 grade of Shanshui Group, the coarse aggregate is granite macadam with the grain size of 5–25 mm, the fine aggregate is river sand produced in Pingdu, Qingdao with fineness modulus of 2.5, the water reducing agent is polycarboxylate superplasticizer with water reducing rate of 25%. The mixing ratio of the original concrete and the compressive strength of the 28 d cube are shown in Table 1.

Cement: Albo 52.5 grade white Portland cement; fine aggregate: river sand produced by Qingdao PingDu with fineness modulus of 2.5; water reducing agent: the use of water reducing rate of 25% polycarboxylic acid water-reducing agent; water: tap water. The matching ratio of RC is shown in Table 2.

### 2.2. Nano-SiO_2_ and CO_2_ Curing of Recycled Coarse Aggregates (RCA)

**Figure 3:**
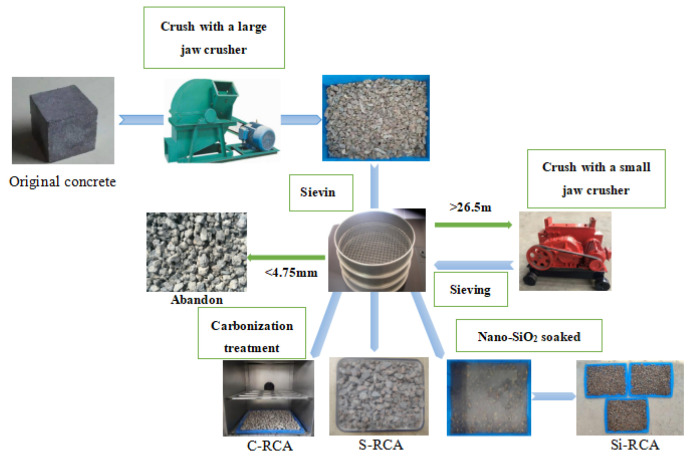
Preparation process of recycled coarse aggregates (RCA): S-RCA–simple crushed of original concrete, C-RCA–simple crushed and carbonization treatment, Si-RCA–simple crushed and nano-SiO_2_ solution soaking.

In this paper, RCA is treated by simple crushing [15], carbonization in carbonization box [16], and soaking in nano-SiO_2_ [17]. Simple crushed RCA (S-RCA) is directly obtained by crushing and sieving the original concrete with a jaw crusher. Firstly, the original concrete was crushed by a large jaw crusher, and the RCA with a particle size between 4.75–26.5 mm was screened by a sieve. Next, a small jaw crusher crushed the RCA with the particle size larger than 26.5 mm for a second time, and then the RCA with the required particle size was screened; The carbonized RCA (C-RCA) was obtained for 72 h in a carbonized chamber with the CO_2_ concentration of (20 ± 3)%, the humidity of (70 ± 5)%, and temperature of (20 ± 2) °C. The carbonized RCA was put in 1% phenolphthalein alcohol solution and carbonization degree was estimated by the color changes of the RCA. The RCA was soaked in 3% nano-SiO_2_ solution for 72 h and dried in the natural state to obtain the RCA (Si-RCA) modified by nano-SiO_2_ soaking. The test was used in nano-SiO_2_ solution produced by Ningbo Bo Wafas Nano Technology Co., Ltd., (Ningbo, China) and the silica mass fraction in the solution was 30%, and the average particle diameter was 10 nm. The soaking method is shown in Figure 3.

### 2.3. Recycled Concrete (RC) Preparation and Aggressive Ions Attack Experiment

According to the mixture ratio in Table 2, 100 × 100 × 100 mm^3^ RC test blocks were prepared by S-RCA, C-RCA, and Si-RCA. After standard curing for 28 d, the RC test blocks were dried to a constant weight. To ensure the one-dimensional transmission of erosion ions, the five faces of the RC test blocks were sealed with paraffin [18]. Then the test blocks were immersed in 3.5% NaCl solution or 5% Na_2_SO_4_ solution for 30 days, respectively. The solution should be replaced every 15 days to ensure that the pH value of the solution remains the same [19].

### 2.4. Macro and Micro Characterization of Recycled Concrete (RC)

#### 2.4.1. Ions Permeability Test

After 30 days of erosion, the paraffin on the surface of the test block was removed with a small blade to test its compressive strength. After suffering from Cl^−^ and SO_4_^2−^ erosion, 10 layers of the eroded surface (each layer with a thickness of 1 mm) of RC was ground by using a concrete layered grinder for ions transmission analysis. According to the Test Rules for Concrete in Water Transportation Engineering (JTJ270-1998) [20], the content of Cl^−^ in RC ground powder was determined by the silver nitrate chemical titration method, and SO_4_^2−^ was determined by the turbidimetric method [21]. Compressive strength and ion erosion concentration were used as macroscopic indexes.

#### 2.4.2. Surface Products Analysis of the Modified Recycled Coarse Aggregates (RCA)

The old mortar attached to the surface of RCA under different repair methods was knocked down with a hammer, then crushed with a mortar and screened with a 240 mesh sieve to obtain samples for X-ray diffraction (XRD, DX-27mini desktop diffractometer, the manufacturer located in Dandong, China) test. The operating power was 600 W (40 kV, 15 mA) and the target samples were scanned over a range of 2θ from 5–80°, with a step size of 0.02° and a scanning speed of 2.5°/min.

#### 2.4.3. Micro-Mechanical Properties and Micro-Morphology Analysis of Recycled Concrete (RC)

The porous and loose ITZs are the main erosion channel for the penetration of aggressive ions. Therefore, the micro-hardness and micromorphology of ITZs were characterized by a micro-hardness tester and scanning electron microscope (SEM). A 100 × 100 × 10 mm^3^ sample of RC was cut with a double-knife precision concrete cutting machine and then ground and polished with a metallographic polishing machine before the test. The polishing procedure was as follows: soak the cut sample in absolute ethyl alcohol for 24 h to replace the free water in the sliced sample, then place the sample in an oven at 50 °C and dry it to constant weight; grind samples with sandpaper of 320 mesh, 800 mesh, 1200 mesh and 1500 mesh in turn on a metallographic grinding machine, grinding each sample for 10–20 min on average according to the grinding effect, lubricating with absolute ethyl alcohol in the grinding process; and finally polish the ground test block with a polishing cloth and a 2.5 μm high-efficiency diamond metallographic polishing agent for 10 min to obtain a micro-hardness test sample.

**Figure 4:**
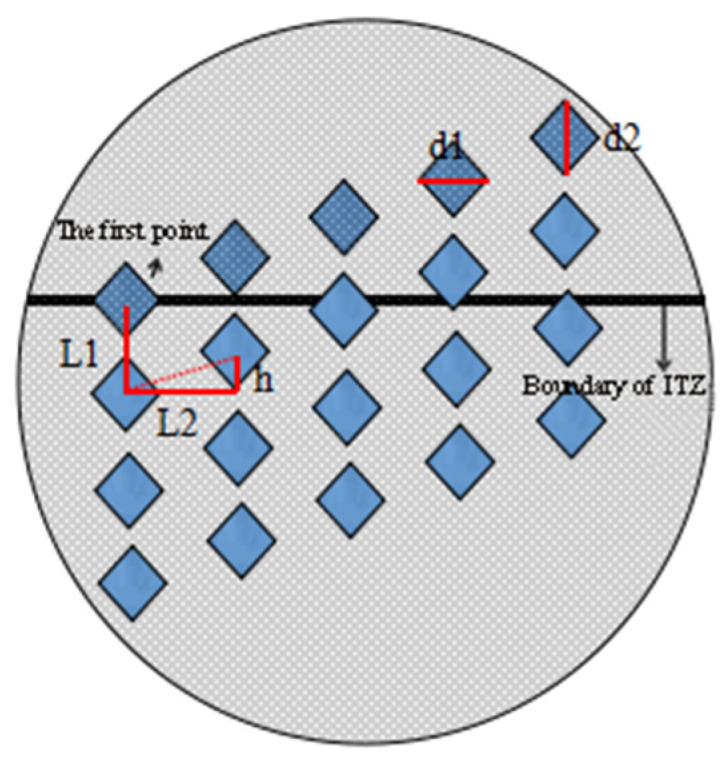
Schematic diagram of micro-hardness tester: d1, d2—diagonal length of indentation at microhardness measuring point, L1–longitudinal spacing of indentation center points, L2–transverse horizontal spacing of indentation center point.

**Figure 5:**
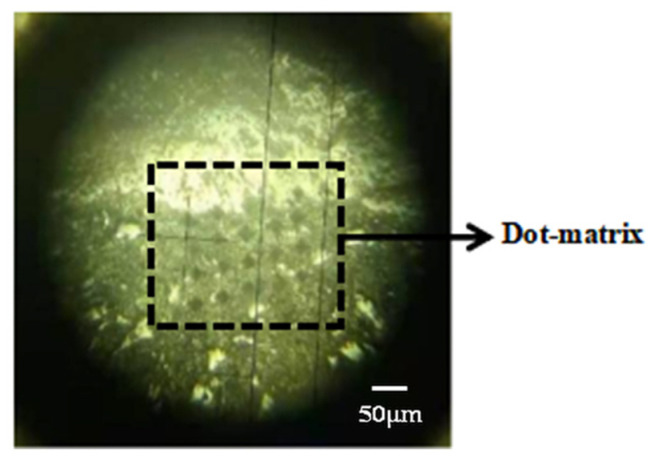
The actual picture after using the micro-hardness tester.

In order to reduce the discreteness of micro-hardness values, firstly, three kinds of ITZs were found near the same aggregate, and secondly, nine lattices were tested in each ITZs, each lattice contains 4 × 5 measuring points. In order to avoid the overlapping phenomenon of adjacent indentations, the test load was determined to be 50 g, and the longitudinal distance L1 and horizontal distance L2 of two adjacent indentations are both 50 μm. In order to visually observe the change of micro-hardness value at the ITZs, the vertical height difference h between two adjacent indentations in the transverse direction was determined to be 10 μm, and we made sure that the first point of the lattice was on the boundary of the interface [22,23]. The schematic diagram of micro-hardness dotting is shown in Figure 4, and the actual dotting diagram is shown in Figure 5.

**Figure 6:**
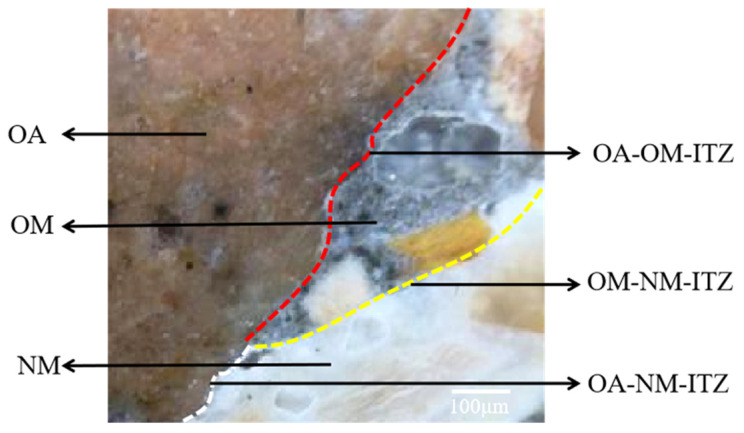
Schematic diagram of interface transition zones (ITZs): OA–order aggregate, OM–order mortar, NM–new mortar, OA-OM-ITZ–the interface between old aggregate and old mortar, OA-NM-ITZ–the interface between old aggregate and new mortar, OM-NM-ITZ–the interface between old mortor and new mortar.

As the old mortar attached by RCA was gray and the RC mixture was white cement, the sample can clearly distinguish the interface between old aggregate and old mortar (OA-OM-ITZ), the interface between old aggregate and new mortar (OA-NM-ITZ), and the interface between old mortar-new mortar (OM-NM-ITZ). Using a Tipscope Microscopic camera as shown in Figure 6, first, a 10 × 10 × 10 mm^3^ concrete sample was cut with a metallographic concrete cutting machine to ensure that the side of the sample contained three kinds of ITZ. Then, all the other sides were sealed with paraffin wax except the observation surface. Finally, the sample was sprayed with gold and vacuumed for SEM (Hitachi, S-3400N) test.

## 3. Results and Discussion

### 3.1. Basic Performance Indexes of Recycled Coarse Aggregates (RCA)

According to the Chinese standard of RCA for Concrete (GB/T25177-2010), the crushing index, water absorption, apparent density, and porosity of S-RCA, C-RCA, and Si-RCA were tested, and the results are shown in Table 3.

As can be seen from Table 3, compared with the controlled group, the crushing index of the RCA treated by carbonization and nano-SiO_2_ soaking decreased by 15.5% and 25.3%, respectively, the water absorption rate decreased by 1% and 9.5%, and the apparent density increased by 1.9% and 1.7%. The above results suggest that both the curing methods can significantly decrease the crushing index of RCA, and it shows slight improvement in the water absorption, apparent density and void fraction. This is because CO_2_ and nano-SiO_2_ reacted with CH in the mortar attached to the surface of the aggregate to generate CaCO_3_ and C–S–H, respectively, thus filling the excessive pores in the mortar attached to the surface of aggregate and improving the quality of RCA [24,25].

**Figure 7:**
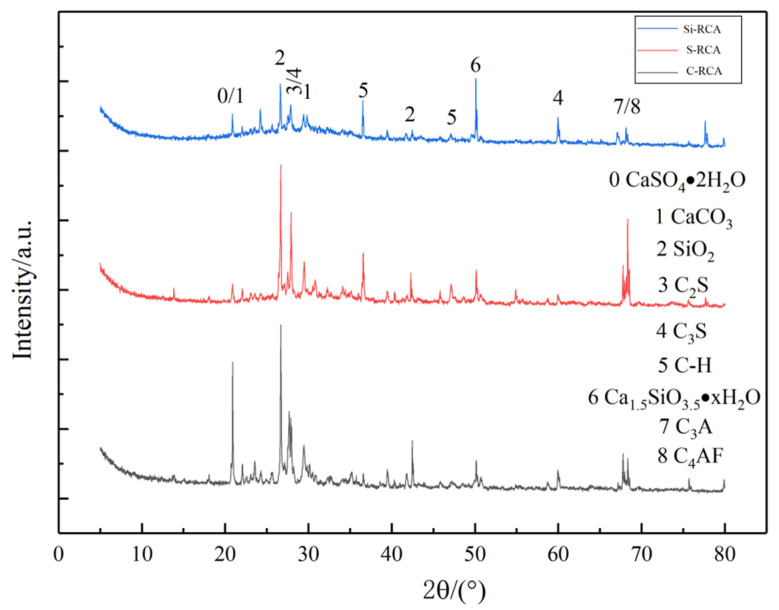
X-ray diffraction (XRD) pattern of RCA before and after modification.

Figure 7 is XRD analysis results of RCA before and after modification. Referring to the existing literature and combining with JADE software to analyze the peak value of the map, the composition of the old mortar was semi-quantitatively analyzed. It can be seen from Figure 7 that the high diffraction peak of SiO_2_ in Si-RCA is due to the adhesion of nano-SiO_2_ on the surface of old aggregate mortar, and the high diffraction peak of SiO_2_ in S-RCA and C-RCA comes from the fine aggregate left in the sample during the sample preparation process. The intensity of CaCO_3_ diffraction peak in C-RCA is higher than that in Si-RCA and S-RCA, which can be attributed to the generated CaCO_3_ via chemical reaction between CO_2_ and CH, C-S-H in old mortar. The diffraction peak of C–S–H is not apparent in this form, but the Ca_15_SiO_3.5_·× H_2_O was generated after crystallization and shows an obvious diffraction peak. Furthermore, the content of Ca_15_SiO_3.5_·× H_2_O in Si-RCA is relatively high because of the chemical reaction between nano-SiO_2_ and CH in old mortar. Compared with the modified aggregates, the S-RCA exhibits higher diffraction peaks of C_2_S and C_3_S, this is probably because CO_2_ and nano-SiO_2_ promote the hydration reaction of C_2_S and C_3_S in the attached old mortar, resulting in the decrease of the two substances.

### 3.2. Macro Performance of Recycled Concrete (RC) after Ion Erosion

**Figure 8:**
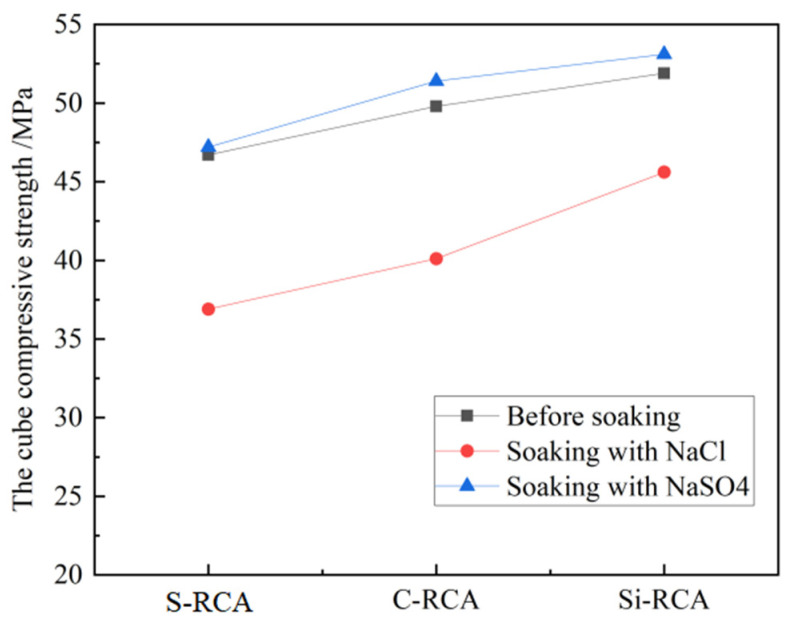
Compressive strength of RC.

Referring to the Chinese Standard for Test Methods of Mechanical Properties of Ordinary Concrete (GB/T50081-2016), the influence of repair methods on the mechanical properties of RC before and after ion erosion was comparatively analyzed, as shown in Figure 8. It can be clearly seen that Cl^−^ erosion has a significant deterioration on RC after soaking for 30 days. After the process of soaking, the compressive strength of RC prepared by S-RCA, C-RCA, and Si-RCA has decreased by 21%, 19.5%, and 12.1%, respectively. Compared with the former, the compressive strength of RC increases slightly after 30 d of SO_4_^2−^ erosion, which may be due to SO_4_^2−^ reacts with CH and C-S-H in concrete to generate a small amount of AFt, which can fill the pores in concrete [26,27] and thus increase the compressive strength. The above results also reveal that nano-SiO_2_ modified RC exhibits a higher compressive strength than CO_2_ cured one.

**Figure 9:**
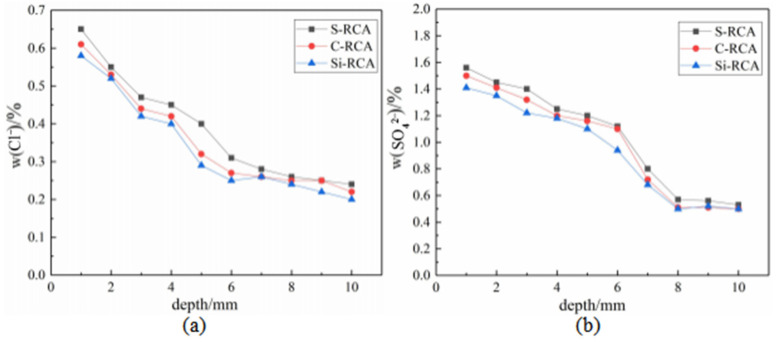
The content of aggressive ions: (**a**)–the content of Cl^−^ in RC, (**b**)–the content of SO_4_^2−^ in RC.

The content of Cl^−^ in RC ground powder was determined by using chemical titration. The test results are shown in Figure 9a. After carbonization and nano-SiO_2_ curing of RCA, the chloride ion permeability resistance of the modified RC is significantly enhanced, and nano-SiO_2_ displays superior effect in repairing RCA. This is because the ITZs inside the concrete are the main channel for the penetration and diffusion of chloride ions, and nano-SiO_2_ can react with the abundant CH at the interface to produce denser C–S–H gel [28], which can densify the unconsolidated ITZs and effectively inhibit the erosion of Cl^−^. In addition, nano-SiO_2_ on the surface of RCA can also enter into the concrete mixture, and react with the hydration products to increase the compactness of concrete. The content of SO_4_^2−^ in RC was measured by turbidimetry, and the results are presented in Figure 9b. The modified RC also exhibits an inhibiting effect of SO_4_^2−^ attack. It is noteworthy that the content of SO_4_^2−^ tends to be stable at the depths exceed 8 mm due to the existence of gypsum in concrete mixing species.

### 3.3. Micro-Performance Analysis of Recycled Concrete (RC) after Ion Erosion

#### 3.3.1. Micromechanical Properties of ITZs in Modified Recycled Concrete (RC) under Different Erosion Environments

**Figure 10:**
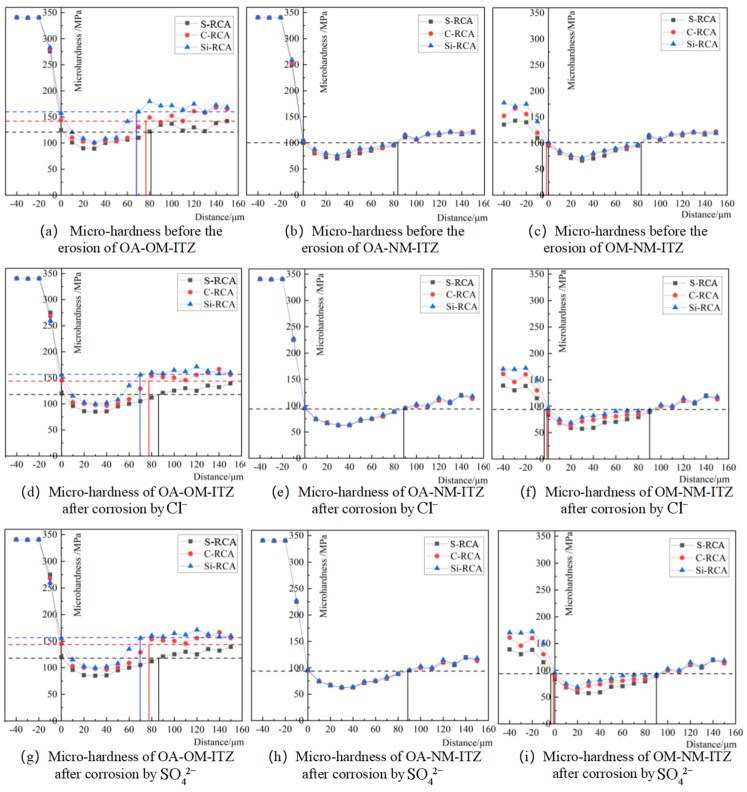
The micro-hardness of the ITZs before and after ions erosion.

The micro-hardness of ITZs of three kinds of RC before and after ion erosion was tested by a micro-hardness tester, and the interface area was quantitatively characterized. The test results are shown in Figure 10. From Figure 10a,d,g, we can clearly see that the micro-hardness of OA-OM-ITZ of modified RC is higher, and the width of the interface zone is smaller than that of concrete prepared from the simple crushed RCA, before and after ion erosion. Before ion erosion, compared with S-RCA recycled concrete, the micro-hardness of OA-OM-ITZ of C-RCA and Si-RCA recycled concrete increased by 7.0–12.0% and 14.2–31.1%, and the width of ITZ decreased by 3.7% and 16.0%; after Cl^-^ erosion, compared with J-RCA recycled concrete, the micro-hardness of OA-OM-ITZ of C-RCA and Si-RCA recycled concrete increased by 13.5–13% and 17.6–29.6%, and the width of ITZ decreased by 9.2% and 19.5%; after SO_4_^2−^ erosion, compared with S-RCA recycled concrete, the micro-hardness of OA-OM-ITZ of C-RCA and Si-RCA recycled concrete increased by 13–9.8% and 23.2–30.1%, and the width of ITZ decreased by 11.4% and 13.9%. Figure 10c,f,i shows the OM-NM-ITZ micro-hardness change law is the same as that of OA-OM-ITZ, which reflects the fact that the interface zone of RC prepared by Si-RCA has better compactness and better resistance to ion erosion. However, the micro-hardnessof the OA-OM-ITZ changes more The micro-hardness value of new mortar is smaller than that of old mortar, and remains the same. This is because carbonization treatment and nano-SiO_2_ soaking mainly act on the old mortar on the RCA, and the mixture ratio of the new mortar of the RC is the same, so the micro-hardness value of the new mortar of the modified RC is almost the same. It can be seen from Figure 10b,e,h that under the same erosion environment, the restoration effect of RCA on OA-NM-ITZ is not apparent, and the width of the interface area is consistent; Comparing ITZs at the same interface under different erosion environments, it can be found that, compared with non-soaked RC, Cl^−^ erosion makes the micro-hardness of each interface zone decrease and the width of the interface zone increase, while SO_4_^2−^ erosion makes the micro-hardness of each interface zone slightly increase and the width of the interface zone decrease. Taking Figure 10b,e,h OA-NM-ITZ as an example, compared with before soaking, the width of the interface zone after Cl^−^ erosion increases by about 7.2%, and that after SO_4_^2−^ erosion decreases by about 4.8%. This is because ITZs are enriched in large amounts of calcium aluminate hydrate and CH [29]. Cl^−^ reacts with calcium aluminate hydrate through the high-porosity ITZ to form Friedel salt, which continues to react with CH to form the diffusely soluble complex CaCl_2_, thus softening ITZs, reducing their hardness, and increasing their width accordingly. On the contrary, due to the short erosion time, SO_4_^2−^ reacts with CH and C-S-H at the interface to form AFt and gypsum, which increase the hardness of ITZs.

#### 3.3.2. Micromorphology of Modified Recycled Concrete (RC)

**Figure 11:**
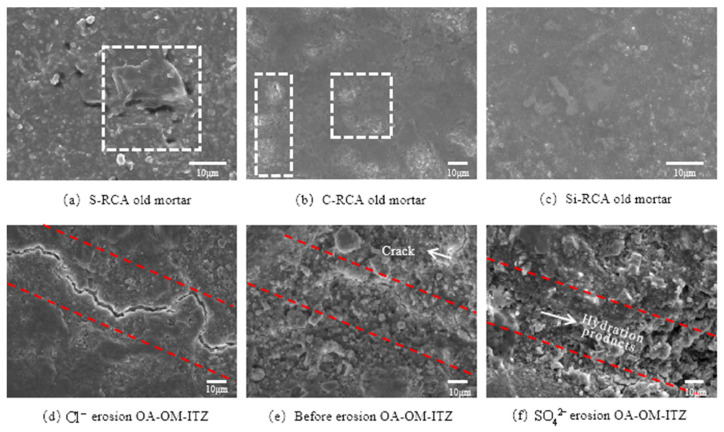
Scanning electron microscopy (SEM) micromorphology.

According to the micro-hardness test results, we found the OA-OM-ITZ in modified RC and the old mortar matrix show higher micro-hardness values. Therefore, the old mortar matrix before ions erosion was characterized by SEM, as shown in Figure 11a–c. It can be seen that the surface of the old mortar matrix of RC prepared by S-RCA exhibits obvious holes and loose microstructure features; The surface of the old mortar matrix under C-RCA and Si-RCA are more compact, flat and without obvious pores. The above results are consistent with the result of high micro-hardness of the modified old mortar matrix. Figure 11d–f are the micro-topography of OA-OM-ITZ of RC prepared by Si-RCA under different erosion environments. It is obvious that the microcrack width in the ITZ is narrow before ions attack. However, a significant increase in the microcrack width of the interface occurred after 30 days’ immersion in chloride solution. Moreover, the content of hydration products and microcrack are decreased, and this may be due to the produced double salts and other products of the reaction between Cl^−^ and CH, which break the balance state of CH and C–S–H gel. As a result, the C–S–H gel continues to decompose, result in the further expansion of microcrack in ITZ. In contrast, after the erosion of SO_4_^2−^ for 30 days, many irregular hydration products are enriched at the interface, and the microcrack becomes unclear (Figure 11f). This is because under the dual action of SO_4_^2−^ and nano-SiO_2_, the micro-cracks and pores are filled, and thus bring about the densification of the interface area.

#### 3.3.3. Elements Distribution of ITZs in Modified Recycled Concrete (RC) under Different Erosion Environments

**Figure 12:**
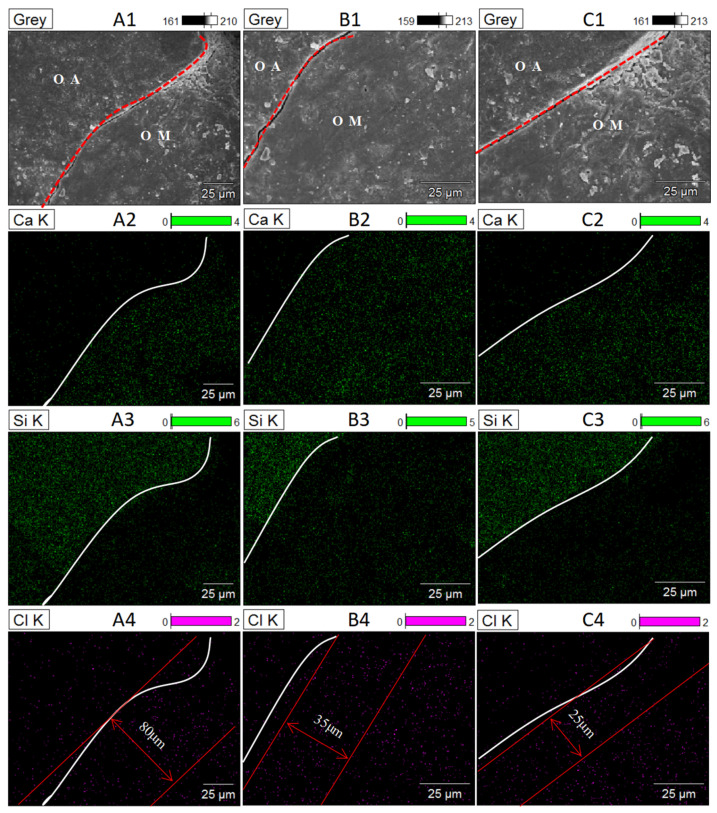
Surface features and the corresponding elemental mapping of OA-OM-ITZ after Cl^−^ attack: (**A_1_**–**A_4_**) S-RCA recycled concrete, (**B_1_**–**B_4_**) C-RCA recycled concrete, (**C_1_**–**C_4_**) Si-RCA recycled concrete.

**Figure 13:**
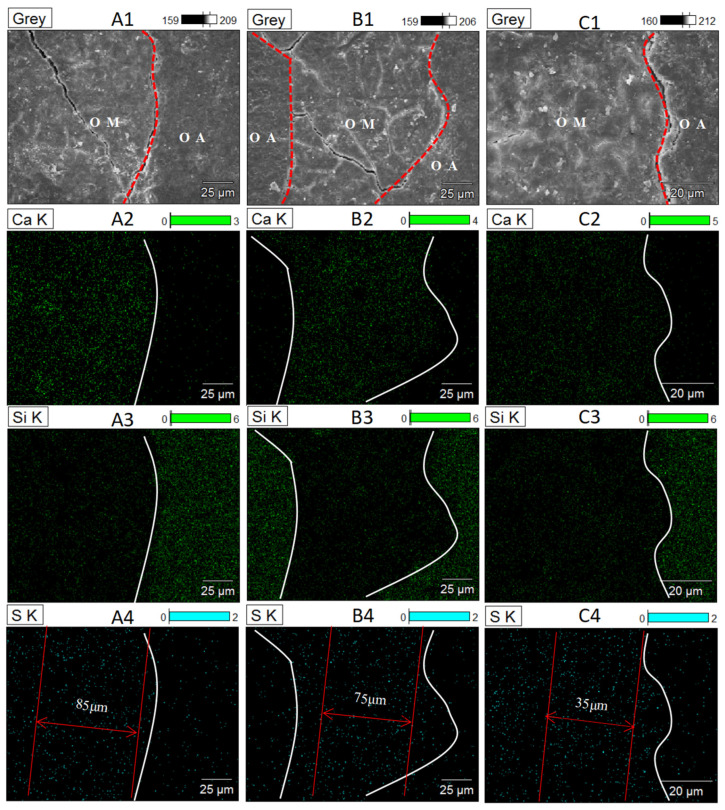
Surface features and the corresponding elemental mapping of OA-OM-ITZ after SO_4_^2−^ attack: (**A_1_**–**A_4_**) S-RCA recycled concrete, (**B_1_**–**B_4_**) C-RCA recycled concrete, (**C_1_**–**C_4_**) Si-RCA recycled concrete.

In order to further interpret the significant effects of aggressive ions on the OA-OM-ITZ of RC, the elements distribution characteristics of Ca, Si, Cl and S in OA-OM-ITZ were ascertained by using an energy-dispersive spectrometer (EDS), as shown in Figure 12 and Figure 13.

Figure 12 shows the element distribution law of Cl^−^ eroded OA-OM-ITZ in RC. According to the distribution law of calcium and silicon, the boundary between aggregate and mortar matrix can be accurately determined. It can be seen from SEM images that there are apparent cracks between the old aggregate matrix and the mortar matrix. According to the Cl^−^ enrichment area, it can be seen that the chloride ion enrichment degree at the interface boundary of S-RCA recycled concrete is relatively high in the range of about 80 μm, because the loose and porous interface area is the principal ion enrichment area, so it can be judged that the width of OA-OM-ITZ of S-RCA recycled concrete is about 80 μm. In the same way, the width of OA-OM-ITZ in C-RCA and Si-RCA prepared RC is about 35 μm and 25 μm, respectively. Although this result is slightly different from that determined by the micro-hardness test, all above results demonstrated that the width of OA-OM-ITZ in RC was reduced after modification. Furthermore, the results reveal that nano-SiO_2_ modification can significantly inhibit the penetration and diffusion of Cl^−^ in the interface zone of RC.

The element distribution law of the OA-OM-ITZ of RC eroded by SO_4_^2−^ is shown in Figure 13. It can be seen from SEM images that there are apparent cracks between aggregate matrix and mortar matrix, and the old mortar matrix of S-RCA recycled concrete contains more micro-cracks than that of modified RC. In the case of elemental mapping of S, we can find that the width of OA-OM-ITZ of S-RCA, C-RCA, and Si-RCA prepared RC is about 85 μm, 75 μm, and 35 μm, respectively. The results are consistent with the micro-hardness test result. Based on the above results, we can infer that the nano-SiO_2_ curing exhibits more superior mechanical performance and durability of RC than carbonation treatment.

### 3.4. Mechanism Analysis

**Figure 14:**
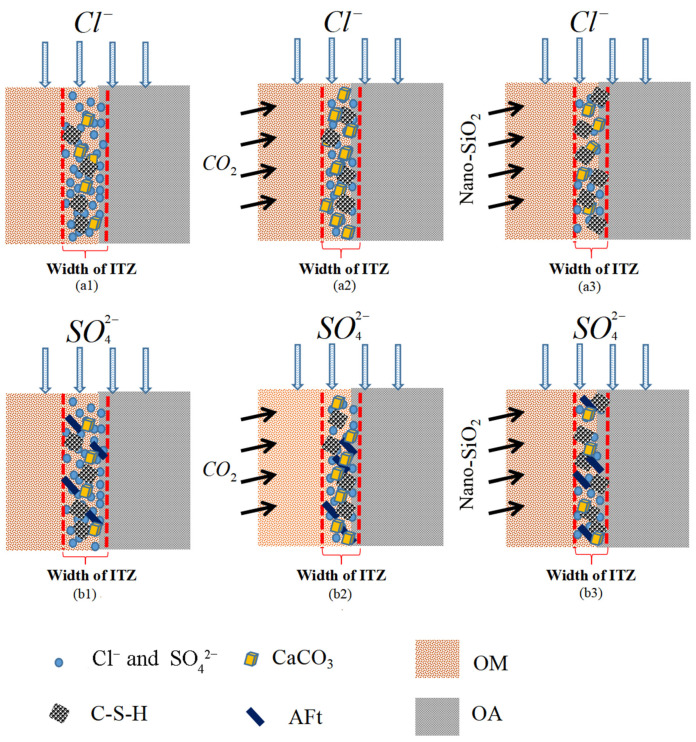
Schematic diagram of influence of aggregate modification on OA-OM-ITZ: (**a1**–**a3**) modified RC under Cl^−^ erosion, (**b1**–**b3**) modified RC under SO_4_^2−^ erosion.

Based on the above results, we propose a basic mechanism model to describe the modification effects of different strategies under ions attack, as shown in Figure 14. For the carbonation treatment, CO_2_ could enter the pore solution through the capillary holes and microcracks of the old mortar, in the presence of water molecules, CO_2_ reacts with cement hydration products and unhydrated cement particles to form CaCO_3_ and silica gel [30,31]. Insoluble CaCO_3_ fills the pores and micro-cracks at OA-OM-ITZ, which increases the density of the interface. As shown in Figure 14(a2,b2), there are more CaCO_3_ at the interface, and related literature studies have found that C–S–H agglomerates more easily around CaCO_3_, thus hindering the further transmission of erosion media. The CH crystal at the interface is large and forms a preferred orientation layer, nano-SiO_2_ reacts with CH to generate C–S–H gel, which promotes the further hydration of cement, as shown in Figure 14(a3,b3) the generated C–S–H gel can not only reduce the porosity of the interface transition zone, but also effectively fill the pores and microcracks in the old mortar matrix.

The form of erosion medium Cl^−^ is mainly divided into two: one exists in free ions, and one is in the form of a combination, wherein the binding form is also divided into physical adsorption and chemical binding. The chlorine elements at the interface are scanned, and it is possible to characterize the presence of free chloride ions and binding chloride ions, and the interface region width is shown in Figure 14(a1–a3). Compared with S-RCA recycled concrete, carbonation treatment and soaking in nano-SiO_2_ make the hydration products at the interface richer, increase the interface density, hinder the further transmission of Cl^−^, and reduce the chlorine enrichment area correspondingly.

SO_4_^2−^ eroded RC mainly includes physical crystal expansion failure and chemical product expansion failure. Most of the aluminum in Portland cement exists in calcium aluminate hydrate and calcium sulphoaluminate hydrate (AFm). With the participation of CH, when RC is in contact with SO_4_^2−^, the two hydration products containing aluminum will be transformed into the high-sulfur hydration product AFt, and the reaction formula is as follows [32]:

$$C_{3}A•CH•H_{18}+2CH+3\bar{S}+11H\to C_{3}A•3C\bar{S}•H_{32}$$

$$C_{3}A•C\bar{S}•H_{18}+2CH+2\bar{S}+12H\to C_{3}A•3C\bar{S}•H_{32}$$

Due to the short soaking time, a small amount of AFt produced by SO_4_^2−^ and hydration products can fill the pores and micro-cracks at the interface. On the basis of CaCO_3_ generated by carbonation-modified RC, the existence of AFt can further improve the interface, as shown in Figure 14(b2). Similarly, on the basis of C–S–H produced by nano-SiO_2_ modified RC, the existence of AFt further improves the performance of interface zone, as shown in Figure 14(b3). Some scholars believe that hydration products can react with sulfate attack medium to form insoluble gypsum [33]. When the attack medium is NaSO_4_ and the attack age is short, NaOH as a by-product of the reaction can ensure the continuity of high alkalinity in a concrete system [34], which plays a vital role in the stability of C–S–H at the interface, and improves the performance of the interface area.

## 4. Conclusions

This paper compared the properties of RC prepared with nano-SiO_2_ and CO_2_ cured RCA subjected to aggressive ions environment, and the main conclusions drawn are summarized below:

Compared with the SO_4_^2−^ erosion, Cl^−^ attack significantly decreases the compressive strength of RC; nevertheless, nano-SiO_2_ curing shows higher compressive strength than carbonation treatment.

According to the micro-hardness test results, Cl^−^ penetration led to the increase of the width of each interface zone and decreases the micro-hardness value. However, the recycled concrete prepared by Si-RCA has the smallest interface area width and the largest micro-hardness value after Cl^−^ and SO_4_^2−^ attack.

The SEM and elemental analysis results of the OA-OM-ITZ in corroded S-RCA and C-RCA show that there are apparent cracks between the old aggregate matrix and the old mortar matrix. However, the nano-SiO_2_ curing can obviously decrease the width and number of cracks in the ITZs, and thus exhibits better erosion resistance to Cl^−^ and SO_4_^2−^. Furthermore, the width of the interface zone obtained from the enrichment areas of Cl and S elements exhibited a consistent trend with the micro-hardness test results.

These findings inferred that nano-SiO_2_ curing exhibits superior mechanical performance and durability of recycled concrete than carbonation treatment under Cl^−^ and SO_4_^2−^ attack due to the more uniform and dense ITZ.

## Figures and Tables

**Table 1:** The original concrete mixture ratio.

Strength Grade	Cement kg/m^3^	Fine Aggregate kg/m^3^	Coarse Aggregate kg/m^3^	W/B	*W* (Water Reducing Agent)%	28 d Compressive Strength MPa
C30	300	780	1170	0.39	1	45.1

**Table 2:** Recycled concrete (RC) mixture ratio.

Strength Grade	White Cement kg/m^3^	Fine Aggregate kg/m^3^	Coarse Aggregate kg/m^3^	W/B	*W* (Water Reducing Agent)%
C45	350	750	980	0.46	1

**Table 3:** Basic performance index of modified Recycled Coarse Aggregates (RCA.)

Types of RCA	Crush Indicators %	Water Absorption %	The Apparent Density kg/m^3^	Void Fraction %
S-RCA	13.27	5.82	2477.17	50.61
C-RCA	11.21	5.76	2523.81	48.28
Si-RCA	9.91	5.27	2519.57	49.08

## Data Availability

The data presented in this study are available on request from the corresponding author.
